# A comparative analysis of three distinct fractional derivatives for a second grade fluid with heat generation and chemical reaction

**DOI:** 10.1038/s41598-024-55059-9

**Published:** 2024-02-23

**Authors:** Sehra, Haleema Sadia, Sami Ul Haq, Hadil Alhazmi, Ilyas Khan, Shafiullah Niazai

**Affiliations:** 1 Shaheed Benazir Bhutto Women University Peshawar, Peshawar, 25000 Khyber Pakhtunkhwa Pakistan; 2 Department of Mathematics, Islamia College Peshawar, Peshawar, 25000 Khyber Pakhtunkhwa Pakistan; 3 Department of Mathematical Sciences, College of Science, Princess Nourah Bint Abdulrahman University, P.O. Box 84428, 11671 Riyadh, Saudi Arabia; 4 Department of Mathematics, College of Education, Majmmah University, 11952 Al-Majmaah, Saudi Arabia; 5 Department of Mathematics, Education Faculty, Laghman University, Mehtarlam City, 2701 Laghman Afghanistan

**Keywords:** Non-Newtonian fluid, Second grade fluid, Fractional operators, CF, CPC, ABC, Mittag Leffler, G-functions, Mathematics and computing, Physics

## Abstract

This article provides a comparison among the generalized Second Grade fluid flow described by three recently proposed fractional derivatives i.e. Atangana Baleanu fractional derivative in Caputo sense (ABC), Caputo Fabrizio (CF) and Constant Proportional-Caputo hybrid (CPC) fractional derivative. The heat mass transfer is observed during the flow past a vertical porous plate that is accelerated exponentially under the effects of the Magneto hydro dynamics. The effects of the heat generation and exponential heating in the temperature boundary layer and chemical reaction at the concentration boundary layer are also analyzed in this article. The flow model is described by three partial differential equations and the set of non-dimensional PDE’s is transformed into ODE’s by utilization of the integral transform technique (Laplace transform). For the better understanding of the rheological properties of the Second Grade fluid we used the CF, ABC and CPC operators to describe the memory effects. The analytical exact solution of the problem is obtained in the form of G-functions and Mittag Leffler functions. For the physical significance of flow parameters, different parameters are graphed. From this analysis it is concluded that the CPC is the most suitable operator to describe the memory effects.

## Introduction

Fluids are classified into two main classes that are the Newtonian fluids and the non-Newtonian fluids. On the basis of the reaction to the shear stresses the non-Newtonian fluids are basically categorized into three classes. 1. Differential-Type fluid, 2. Rate-type fluid and 3. Integral-type fluid. Differential-type fluids are those whose shear strain and shear rate are related to each other. Rate type fluids are those fluids with visco-elastic properties. Integral type fluids are those whose Shear rate barely influences shear stress. Among the non-Newtonian fluid Models, the second-grade fluid model is the most commonly used model and belongs to the differential type non-Newtonian fluid Models.

Systematized investigation of these fluid models is important for practical implementations and theoretical studies in the manufacture of modern machinery. Based around the idea of the fluid mechanics, different aspects of second grade fluid was explored by re-searchers, scientists, engineers and mathematicians dependent upon different situation. Many scientists and researchers are concentrated to examine the geometrical flow scheme of Second-Grade fluid for configuration of some important characteristics due to wide range of theoretical and real life applications. For example; in^1–5^ the researchers computed the analytical solutions for the second grade unsteady flow by making use of the method of variables separation. The authors in^6^ presented a second-grade fluid model to study the influences of heat flux with radiation and MHD, and calculated the analytical solution for the problem by using Laplace integral transform. Some important aspects of second grade fluid are interpreted in^7–11^.

The importance of the exact or numerical solution in the real life is the main reason for its exploration. In order to find the exact solution many mathematicians, investigator and researchers used many methods, for instance multi step approach^12,13^, unified method^14^, reproducing the kernel Hilbert space method^15,16^ (RB- SODET)^17^, residual power series method^18^ and simple equation modification method^19^. The authors applied the Caputo derivative for the qualitative analysis of the grade second fluid and achieved the analytical exact solution of the flow scheme in^20^. The authors examined the impacts of the heat source on the Second-Grade fluid flowing with influence of heat mass transfer and magneto hydro dynamics over an exponentially accelerated surface in porous medium by using Prabhakar fractional derivative^21^.

The authors used the fractal two scale techniques and energy balanced method for the approximation of the exact analytical solution in^22^. In^23^ the authors used the Caputo Fabrizio (CF) time derivative of fractional order for the analysis of the free convection heat mass transfer of nano-particles with base fluid “water” with ramped wall condition. In^24,25^ the authors described the generalized fractional flow of differential-type fluid by using two different fractional derivative i.e. the CF (Caputo Fabrizio) and AB (Atangana Baleanu) fractional derivative and obtained the exact analytical result for the problem. But all these considered problems are without the consideration of mass transfer.

In this study, exact solutions for the rotational flow of a fractional Oldroyd-B fluid within an annulus are obtained through the application of integral transforms^26^. The authors created a framework which highlights the significance of studying a two-dimensional, steady, and incompressible axisymmetric flow of a Maxwell hybrid nanofluid between double disks, incorporating the influence of thermal radiation^27^. The authors employed the modified Khater technique with conformable fractional derivatives and the Adomian decomposition method to investigate analytical and semi-analytical wave solutions for the perturbed time-fractional nonlinear Schrodinger (NLS) problem^28^. The authors investigated numerical solutions for the nonlinear fractional Ostrovsky equation using five modern numerical methods: Adomian decomposition (AD), El Kalla (EK), Cubic B-Spline (CBS), extended Cubic B-Spline (ECBS), and exponential Cubic B-Spline (ExCBS)^29^.

In the available published literature the effects of the mass transfer, heat generation, chemical reaction and the comparative analysis of the three fractional derivative operators ABC, CF and CPC fractional operator with the corresponding initial and boundary conditions and MHD effects in porous medium are neither analyzed nor published. In order to fill this gap we addressed the problem in the present article and obtain the exact analytical solution of the problem in the form of Mittag–Leffler and G-function by using the technique of the integral transform (Laplace transform). For the physical significance of flow parameters, different parameters are graphed. From this analysis it is noticed that the CPC fractional derivative is the most suitable operator to describe the memory effects.

## Mathematical model

Let’s consider second grade fluid with MHD effects flowing via an oscillating (in its respective plane) vertical plate planted into a porous medium. The plate via which the fluid is observe at y = 0 and the flow of fluid is along the vertical plate restrained at y > 0. In the start for ε = 0 (time) the flowing fluid and the plate via which fluid is flowing both are considered to be stationary with ambient/initial temperature of the fluid $\vartheta_{\infty }$ and ambient/initial concentration of the fluid $C_{\infty }$. Shortly afterward when time $\varepsilon = 0^{ + }$, the plate which is initially considered as static starts oscillation and the fluid also starts movement with velocity $u_{0} e^{\omega \varepsilon }$ and concentration becomes $C_{w}$ and temperature $\vartheta_{w}$. The governing equations for second grade fluid flow with MHD effects under Boussinesq's approximation are obtained as in^24,25^.

1 $$\frac{\partial w(y,\varepsilon )}{{\partial \varepsilon }} = \upsilon \left( {1 + \frac{{\alpha_{1} }}{\mu }\frac{\partial }{\partial \varepsilon }} \right)\frac{{\partial w^{2} (y,\varepsilon )}}{{\partial y^{2} }} - \left( {\frac{{\sigma_{0} M_{0}^{2} }}{\rho } + \frac{\upsilon \phi }{{k_{0} }}\left( {1 + \frac{{\alpha_{1} }}{\mu }\frac{\partial }{\partial \varepsilon }} \right)} \right)u(y,\varepsilon ) + g\beta_{\vartheta } \left( {\vartheta \left( {y,\varepsilon } \right) - \vartheta_{\infty } } \right) + g\beta_{C} \left( {C\left( {y,\varepsilon } \right) - C_{\infty } } \right),$$

2 $$\frac{\partial \vartheta (y,\varepsilon )}{{\partial \varepsilon }} = \frac{k}{{\rho C_{p} }}\frac{{\partial \vartheta^{2} (y,\varepsilon )}}{{\partial y^{2} }} - Q_{0} \left( {\vartheta \left( {y,\varepsilon } \right) - \vartheta_{\infty } } \right){, }$$

3 $$\frac{\partial C(y,\varepsilon )}{{\partial \varepsilon }} = D_{m} \frac{{\partial C^{2} (y,\varepsilon )}}{{\partial y^{2} }} - J\left( {C\left( {y,\varepsilon } \right) - C_{\infty } } \right){.}$$

where $\vartheta$(y, $\varepsilon$), C(y, $\varepsilon$), w(y, $\varepsilon$), $\rho , \, \beta_{\vartheta } , \, \beta_{C} , \, \alpha_{1} \, and{\text{ C}}_{p} \,$ are the fluid temperature, fluid concentration, fluid velocity, fluid density, volumetric thermal expansion coefficient, volumetric mass expansion coefficient, second grade parameter and specific heat capacity.

The suitable conditions ICs (initial) and BCs (boundary) are presented as^24,25^:

4 $$\left. \begin{gathered} w(y,0) = 0, \;\;\;\;\;\;\;\;\;\;\;\vartheta (y,0) = \vartheta_{\infty } , \, C(y,0) = C_{\infty } , \, \frac{\partial w(y,\varepsilon )}{{\partial \varepsilon }} = 0,\;\;\;\;\;y \ge 0 \hfill \\ w(0,\varepsilon ) = u_{0} e^{\omega \varepsilon } ,\; \, \vartheta (0,\varepsilon ) = \vartheta_{w} (1 - ae^{ - b\varepsilon } ) + \vartheta_{\infty } ,\;\;\;\;\;C(0,\varepsilon ) = C_{\infty } + \left( {C_{w} - C_{\infty } } \right)e^{\omega \varepsilon } \, a,b \ge 0,\varepsilon > 0 \hfill \\ w(y,\varepsilon ) \to 0, \, \vartheta (y,\varepsilon ) \to \vartheta_{\infty } , \, C(y,\varepsilon ) \to C_{\infty } \, as\; \, y \to \infty . \hfill \\ \end{gathered} \right\}$$

Writing the scheme of considered problem in non-dimensional form, by using non-dimensional quantities/variables are:

5 $$\left. \begin{gathered} w^{*} = \frac{w}{{U_{0} }}, \, y^{*} = \frac{{yU_{0} }}{\nu }, \, \varepsilon^{*} = \frac{{U_{0}^{2} }}{\nu }, \, \theta = \frac{{\vartheta - \vartheta_{\infty } }}{{\vartheta_{w} }}, \, C^{*} = \frac{{C - C_{\infty } }}{{C_{w} - C_{\infty } }}, \hfill \\ Gr = \frac{{g\beta_{\vartheta } \nu (\vartheta_{w} - \vartheta_{\infty } )}}{{u_{0}^{3} }}, \, Gm = \frac{{g\beta_{c} \nu (C_{w} - C_{\infty } )}}{{u_{0}^{3} }}, \, \Pr = \frac{{\mu C_{p} }}{k}, \, b_{0} = \frac{{\alpha_{2} }}{K}, \hfill \\ \eta_{2} = \frac{J\nu }{{u_{0}^{2} }}, \, Sc = \frac{\nu }{{D_{m} }}, \, \alpha_{2} = \frac{{\alpha_{1} u_{0}^{2} \rho }}{{\mu^{2} }}, \, \eta_{1} = \frac{Q\nu }{{u_{0}^{2} }}, \, a_{0} = M + \frac{1}{K}{. } \hfill \\ \end{gathered} \right\}$$

After the implementation of Eq. (5) into Eqs. (1–3), then the dimensionless governing equations of the model are presented as:

6 $$\frac{\partial w(y,\varepsilon )}{{\partial \varepsilon }} = \left( {1 + \alpha_{2} \frac{\partial }{\partial \varepsilon }} \right)\frac{{\partial w^{2} (y,\varepsilon )}}{{\partial y^{2} }} - \left( {M + \frac{1}{K}\left( {1 + \alpha_{2} \frac{\partial }{\partial \varepsilon }} \right)} \right)w(y,\varepsilon ) + G_{r} \vartheta \left( {y,\varepsilon } \right) + G_{m} C\left( {y,\varepsilon } \right){,}$$

7 $$\frac{\partial \vartheta (y,\varepsilon )}{{\partial \varepsilon }} = \frac{{1}}{\Pr }\frac{{\partial \vartheta^{2} (y,\varepsilon )}}{{\partial y^{2} }} - \eta_{1} \vartheta \left( {y,\varepsilon } \right){,}$$

8 $$\frac{\partial C(y,\varepsilon )}{{\partial \varepsilon }} = \frac{1}{Sc}\frac{{\partial C^{2} (y,\varepsilon )}}{{\partial y^{2} }} - \eta_{2} C\left( {y,\varepsilon } \right){.}$$

The IC’s (initial) and BC’s (boundary) conditions in non-dimensional form are;

9 $$\left. \begin{gathered} w(y,0) = 0, \;\;\;\;\;\;\;\;\;\;\;\vartheta (y,0) = 0, \, C(y,0) = 0, \, \;\;\;\;\;y \ge 0 \hfill \\ w(0,\varepsilon ) = e^{\omega \varepsilon } ,\; \, \vartheta (0,\varepsilon ) = 1 - ae^{ - b\varepsilon } ,\;\;\;\; \, C(0,\varepsilon ) = e^{\omega \varepsilon } \, a,b \ge 0,\varepsilon > 0 \hfill \\ w(y,\varepsilon ) \to 0, \, \vartheta (y,\varepsilon ) \to 0, \, C(y,\varepsilon ) \to 0 \, as\; \, y \to \infty . \hfill \\ \end{gathered} \right\}$$

The generalized governing equations for concentration, energy and momentum distributions are designed by using three different fractional derivative operators are described as:

The generalized model for CPC fractional operator is interpreted as:

10 $${}^{CPC}D_{\varepsilon }^{\alpha } u(y,\varepsilon ) = \left( {1 + \alpha_{{_{2} }} {}^{CPC}D_{\varepsilon }^{\alpha } } \right)\frac{{\partial w^{{^{2} }} (y,\varepsilon )}}{{\partial y^{{^{2} }} }} - \left( {M + \frac{1}{K}\left( {1 + \alpha_{{_{2} }} {}^{CPC}D_{\varepsilon }^{\alpha } } \right)} \right)w(y,\varepsilon ) + G_{r} \vartheta \left( {y,\varepsilon } \right) + G_{m} C\left( {y,\varepsilon } \right){,}$$

11 $${}^{CPC}D_{\varepsilon }^{\alpha } \vartheta (y,\varepsilon ) = \frac{{1}}{\Pr }\frac{{\partial \vartheta^{{^{2} }} (y,\varepsilon )}}{{\partial y^{{^{2} }} }} - \eta_{1} \vartheta \left( {y,\varepsilon } \right){,}$$

12 $${}^{CPC}D_{\varepsilon }^{\alpha } C(y,\varepsilon ) = \frac{1}{Sc}\frac{{\partial C^{{^{2} }} (y,\varepsilon )}}{{\partial y^{{^{2} }} }} - \eta_{2} C\left( {y,\varepsilon } \right){.}$$

The generalized model for CF fractional operator is interpreted as:

13 $${}^{CF}D_{\varepsilon }^{\alpha } w(y,\varepsilon ) = \left( {1 + \alpha_{{_{2} }} {}^{CF}D_{\varepsilon }^{\alpha } } \right)\frac{{\partial w^{{^{2} }} (y,\varepsilon )}}{{\partial y^{{^{2} }} }} - \left( {M + \frac{1}{K}\left( {1 + \alpha_{{_{2} }} {}^{CF}D_{\varepsilon }^{\alpha } } \right)} \right)w(y,\varepsilon ) + G_{r} \vartheta \left( {y,\varepsilon } \right) + G_{m} C\left( {y,\varepsilon } \right){,}$$

14 $${}^{CF}D_{\varepsilon }^{\alpha } \vartheta (y,\varepsilon ) = \frac{{1}}{\Pr }\frac{{\partial \vartheta^{{^{2} }} (y,\varepsilon )}}{{\partial y^{{^{2} }} }} - \eta_{1} \vartheta \left( {y,\varepsilon } \right){,}$$

15 $${}^{CF}D_{\varepsilon }^{\alpha } C(y,\varepsilon ) = \frac{1}{Sc}\frac{{\partial C^{{^{2} }} (y,\varepsilon )}}{{\partial y^{{^{2} }} }} - \eta_{2} C\left( {y,\varepsilon } \right){.}$$

The generalized model for ABC fractional operator is interpreted as:

16 $${}^{ABC}D_{\varepsilon }^{\alpha } w(y,\varepsilon ) = \left( {1 + \alpha_{{_{2} }} {}^{ABC}D_{\varepsilon }^{\alpha } } \right)\frac{{\partial w^{{^{2} }} (y,\varepsilon )}}{{\partial y^{{^{2} }} }} - \left( {M + \frac{1}{K}\left( {1 + \alpha_{{_{2} }} {}^{ABC}D_{\varepsilon }^{\alpha } } \right)} \right)w(y,\varepsilon ) + G_{r} \vartheta \left( {y,\varepsilon } \right) + G_{m} C\left( {y,\varepsilon } \right){,}$$

17 $${}^{ABC}D_{\varepsilon }^{\alpha } \vartheta (y,\varepsilon ) = \frac{{1}}{\Pr }\frac{{\partial \vartheta^{{^{2} }} (y,\varepsilon )}}{{\partial y^{{^{2} }} }} - \eta_{1} \vartheta \left( {y,\varepsilon } \right){,}$$

18 $${}^{ABC}D_{\varepsilon }^{\alpha } C(y,\varepsilon ) = \frac{1}{Sc}\frac{{\partial C^{{^{2} }} (y,\varepsilon )}}{{\partial y^{{^{2} }} }} - \eta_{2} C\left( {y,\varepsilon } \right){.}$$

**Fractional derivatives and their Laplace transform:**

1. ${}^{CPC}D_{\varepsilon }^{\alpha } f(y,\varepsilon )$ is the representational form of the CPC fractional operator and its definition is given as:

19 $${}^{CPC}D_{\varepsilon }^{\alpha } f(y,\varepsilon ) = \frac{1}{\Gamma (1 - \alpha )}\int\limits_{0}^{t} {\left( {k_{1} (\alpha )f(y,\tau ) + k_{0} (\alpha )\frac{\partial f(y,\tau )}{{\partial \tau }}} \right)\left( {\varepsilon - \tau } \right)^{ - \alpha } d\tau { 0 < }\alpha { < 1}} {,}$$

After applying the Laplace transformation to the CPC fractional operator is represented as:

20 $$L\left\{ {{}^{CPC}D_{\varepsilon }^{\alpha } f(y,\varepsilon )} \right\} = \left[ {\frac{{k_{1} (\alpha )}}{q} + k_{0} (\alpha )} \right]q^{\alpha } L\left\{ {f\left( {y,\varepsilon } \right)} \right\} - k_{0} (\alpha )q^{\alpha - 1} f\left( {y,0} \right){.}$$

where $\alpha$ represents fractional parameter of CPC time fractional operator.

2. ${}^{CF}D_{\varepsilon }^{\alpha } f(y,\varepsilon )$ is the representational form of the CF fractional operator and its definition is given as:

21 $${}^{CF}D_{\varepsilon }^{\alpha } f(y,\varepsilon ) = \frac{1}{1 - \alpha }\int\limits_{0}^{t} {\exp \left( { - \frac{\alpha (\alpha - \varepsilon )}{{1 - \alpha }}} \right)\frac{\partial f(y,\tau )}{{\partial \tau }}d\tau { 0 < }\alpha { < 1}} {,}$$

After applying the Laplace transformation to the CF fractional operator is represented as:

22 $$L\left\{ {{}^{CF}D_{\varepsilon }^{\alpha } f(y,\varepsilon )} \right\} = \frac{{qL\left\{ {f\left( {y,\varepsilon } \right)} \right\} - f\left( {y,0} \right)}}{(1 - \alpha )q + \alpha }. \,$$

where $\alpha$ represents parameter of CF fractional operator.

3. ${}^{ABC}D_{\varepsilon }^{\alpha } f(y,\varepsilon )$ is the representational form of the ABC fractional operator and its definition is given as:

23 $${}^{ABC}D_{\varepsilon }^{\alpha } f(y,\varepsilon ) = \frac{1}{1 - \alpha }\int\limits_{0}^{t} {E_{\alpha } \left( { - \frac{\alpha (\alpha - \varepsilon )}{{1 - \alpha }}} \right)\frac{\partial f(y,\tau )}{{\partial \tau }}d\tau { 0 < }\alpha { < 1}} ,$$

After applying the method of Laplace transformation to the AB fractional operator is given as:

24 $$L\left\{ {{}^{ABC}D_{\varepsilon }^{\alpha } f(y,\varepsilon )} \right\} = \frac{{q^{\alpha } L\left\{ {f\left( {y,\varepsilon } \right)} \right\} - q^{\alpha - 1} f\left( {y,0} \right)}}{{(1 - \alpha )q^{\alpha } + \alpha }}.$$

where $\alpha$ is the ABC’s fractional parameter.

## Problem solution

In this section, the exact analytical solution achieved by applying the technique of Laplace transformation to the fractionalized second grade fluid model is presented.

### Solution of concentration field

#### By Caputo Fabrizio fractional derivative

After taking the integral transform method (Laplace) to Eq. (15) with the corresponding conditions (IC’s and BC’s) given in Eq. (9), we have;

$$\frac{{\partial^{{^{2} }} \overline{C}(y,q)}}{{\partial^{{^{2} }} y}} - \left( {Sc\left( {\frac{q}{(1 - \alpha )q + \alpha } + \eta_{2} } \right)} \right)\overline{C}(y,q) = 0,$$

With the conditions after taking Laplace transform;

$$\overline{C}(y,q) \to 0{\text{ as y}} \to \infty {\text{ and }}\overline{C}(0,q) = \frac{1}{q - \omega }$$

The transformed solution obtained for the mass concentration field is interpreted as:

$$\overline{C}(y,q) = \frac{1}{q - \omega }\exp \left( { - y\sqrt {Sc\left( {\frac{q}{(1 - \alpha )q + \alpha } + \eta_{2} } \right)} } \right)$$

The equivalent series representation of the solution of the concentration field is represented as;

25 $$\overline{C}(y,q) = \sum\limits_{l = 0}^{\infty } {\sum\limits_{m = 0}^{\infty } {\sum\limits_{n = 0}^{\infty } {\frac{{\left( y \right)^{ - l} \left( {\eta_{{_{2} }} Sc} \right)^{{\frac{l}{2} - m}} \left( {Sc} \right)^{m} \left( \alpha \right)^{n} \Gamma \left( {\frac{l}{2} + 1} \right)\Gamma \left( {m + n} \right)}}{{l!m!n!\left( {1 - \alpha } \right)^{m + n} \Gamma \left( {\frac{l}{2} - m + 1} \right)\Gamma \left( m \right)}}} } } \cdot \frac{1}{{q^{1 + n} }},$$

For the purpose of obtaining the final result of the concentration field, taking the inverse integral transformation (Laplace transform) of the Eq. (25), we get;

26 $$C(y,\varepsilon ) = \sum\limits_{l = 0}^{\infty } {\sum\limits_{m = 0}^{\infty } {\sum\limits_{n = 0}^{\infty } {\frac{{\left( y \right)^{ - l} \left( {\eta_{2} Sc} \right)^{{\frac{l}{2} - m}} \left( {Sc} \right)^{m} \left( \alpha \right)^{n} \Gamma \left( {\frac{l}{2} + 1} \right)\Gamma \left( {m + n} \right)}}{{l!m!n!\left( {1 - \alpha } \right)^{m + n} \Gamma \left( {\frac{l}{2} - m + 1} \right)\Gamma \left( m \right)}}} } } \cdot \frac{{\varepsilon^{n} }}{{\Gamma \left( {1 + n} \right)}},$$

#### By Atangana Baleanu fractional derivative

After taking the integral transforms technique (Laplace technique) to Eq. (18) with the corresponding IC’s and BC’s conditions described in Eq. (9), we have;

$$\frac{{\partial^{{^{2} }} \overline{C}(y,q)}}{{\partial^{{^{2} }} y}} - \left( {Sc\left( {\frac{{q^{{^{\alpha } }} }}{{(1 - \alpha )q^{{^{\alpha } }} + \alpha }} + \eta_{2} } \right)} \right)\overline{C}(y,q) = 0,$$

With the transformed conditions after the Laplace transform as;

$$\overline{C}(y,q) \to 0{\text{ as y}} \to \infty {\text{ and }}\overline{C}(0,q) = \frac{1}{q - \omega }.$$

The transformed solution obtained for mass concentration field is interpreted as:

27 $$\overline{C}(y,q) = \frac{1}{q - \omega }\exp \left( { - y\sqrt {Sc\left( {\frac{{q^{{^{\alpha } }} }}{{(1 - \alpha )q^{{^{\alpha } }} + \alpha }} + \eta_{2} } \right)} } \right),$$

The equivalent series representation of the solution of the concentration field is represented as;

28 $$\overline{C}(y,q) = \sum\limits_{l = 0}^{\infty } {\sum\limits_{m = 0}^{\infty } {\sum\limits_{n = 0}^{\infty } {\frac{{\left( y \right)^{ - l} \left( {\eta_{{_{2} }} Sc} \right)^{{\frac{l}{2} - m}}  \left( {Sc} \right)^{m} \left( \alpha \right)^{n} \Gamma \left( {\frac{l}{2} + 1} \right)\Gamma \left( {m + n} \right)}}{{l!m!n!\left( {1 - \alpha } \right)^{m + n} \Gamma \left( {\frac{l}{2} - m + 1} \right)\Gamma \left( m \right)}}} } } \cdot \frac{1}{{q^{n\alpha + 1} }},$$

For the purpose of obtaining the final result of the concentration field, taking the inverse integral transform (Laplace technique) of the Eq. (28), we get;

29 $$C(y,\varepsilon ) = \sum\limits_{l = 0}^{\infty } {\sum\limits_{m = 0}^{\infty } {\sum\limits_{n = 0}^{\infty } {\frac{{\left( y \right)^{ - l} \left( {\eta_{{_{2} }} Sc} \right)^{{\frac{l}{2} - m}} \left( {Sc} \right)^{m} \left( \alpha \right)^{n} \Gamma \left( {\frac{l}{2} + 1} \right)\Gamma \left( {m + n} \right)}}{{l!m!n!\left( {1 - \alpha } \right)^{m + n} \Gamma \left( {\frac{l}{2} - m + 1} \right)\Gamma \left( m \right)}}} } } \cdot \frac{{\varepsilon^{n\alpha } }}{{\Gamma \left( {n\alpha + 1} \right)}}{.}$$

#### By constant proportional-caputo hybrid fractional derivative

After applying the integral transform technique (Laplace technique) to Eq. (12) with the corresponding conditions (IC’s and BC’s) given in Eq. (9), we have;

$$\frac{{\partial^{{^{2} }} \overline{C}(y,q)}}{{\partial^{{^{2} }} y}} - \left( {Sc\left( {q^{{^{\alpha } }} \left[ {\frac{{k_{{_{1} }} (\alpha )}}{q} + k_{{_{0} }} (\alpha )} \right] + \eta_{2} } \right)} \right)\overline{C}(y,q) = 0,$$

With the IC’s and BC’s (conditions) changed by Laplace transform as;

$$\overline{C}(y,q) \to 0{\text{ as y}} \to \infty {\text{ and }}\overline{C}(0,q) = \frac{1}{q - \omega }.$$

The transformed solution obtained for mass concentration field is interpreted as:

30 $$\overline{C}(y,q) = \frac{1}{q - \omega }\exp \left( { - y\sqrt {Sc\left( {q^{{^{\alpha } }} \left[ {\frac{{k_{{_{1} }} (\alpha )}}{q} + k_{{_{0} }} (\alpha )} \right] + \eta_{2} } \right)} } \right),$$

The equivalent series representation of the solution of the concentration field is represented as;

31 $$\overline{C}(y,q) = \sum\limits_{l = 0}^{\infty } {\sum\limits_{m = 0}^{\infty } {\frac{{\left( y \right)^{ - 1} \left( {\eta_{{_{2} }} Sc} \right)^{{^{{\frac{l}{2} - m}} }} \left( {Sck_{{_{0} }} (\alpha )} \right)^{{^{m} }} \Gamma \left( {\frac{l}{2} + 1} \right)}}{{l!m!\Gamma \left( {\frac{l}{2} - m + 1} \right)}}} } \cdot \frac{{q^{{^{m(\alpha - 1) - 1} }} }}{{(q + d)^{ - m} }},$$

For the purpose of obtaining the final result (exact analytical) solution of the concentration field, after applying the inverse integral transform (Laplace) of the Eq. (26), we get;

32 $$C(y,\varepsilon ) = \sum\limits_{l = 0}^{\infty } {\sum\limits_{m = 0}^{\infty } {\frac{{\left( y \right)^{ - 1} \left( {\eta_{{_{2} }} Sc} \right)^{{\frac{l}{2} - m}} \left( {Sck_{{_{0} }} (\alpha )} \right)^{m}\Gamma \left( {\frac{l}{2} + 1} \right)}}{{l!m!\Gamma \left( {\frac{l}{2} - m + 1} \right)}}} } \cdot G_{{_{1, \, m(\alpha - 1) - 1, \, - m} }} \left( { - d,\varepsilon } \right).$$

### Solution of temperature field

#### By Caputo Fabrizio fractional derivative

After applying the integral transformation technique (Laplace transform) to Eq. (12) with corresponding conditions given in Eq. (9), we have;

$$\frac{{\partial^{{^{2} }} \overline{\vartheta }(y,q)}}{{\partial^{{^{2} }} y}} - \left( {\Pr \left( {\frac{q}{(1 - \alpha )q + \alpha } + \eta_{1} } \right)} \right)\overline{\vartheta }(y,q) = 0,$$

With transformed conditions after taking Laplace transform as;

$$\vartheta (y,q) \to 0{\text{ as y}} \to \infty {\text{ and }}\overline{\vartheta }(0,q) = \frac{1}{q} - \frac{a}{q - b}.$$

The transformed solution obtained for the Temperature field is interpreted as:

33 $$\overline{\vartheta }(y,q) = \left( {\frac{1}{q} - \frac{a}{q + b}} \right)\exp \left( { - y\sqrt {\Pr \left( {\frac{q}{(1 - \alpha )q + \alpha } + \eta_{1} } \right)} } \right),$$

The equivalent series representation of the solution of the temperature field is represented as;

34 $$\overline{\vartheta }(y,q) = \left( {\frac{1}{q} - \frac{a}{q + b}} \right)\sum\limits_{l = 0}^{\infty } {\sum\limits_{m = 0}^{\infty } {\sum\limits_{n = 0}^{\infty } {\frac{{\left( y \right)^{ - l} \left( {\eta_{{_{1} }} \Pr } \right)^{{\frac{l}{2} - m}}\left( {\Pr } \right)^{m} \left( \alpha \right)^{n} \Gamma \left( {\frac{l}{2} + 1} \right)\Gamma \left( {m + n} \right)}}{{l!m!n!\left( {1 - \alpha } \right)^{m + n}\Gamma \left( {\frac{l}{2} - m + 1} \right)\Gamma \left( m \right)}}} } } \cdot \frac{1}{{q^{n\alpha + 1} }},$$

For the purpose of obtaining the targeted result (exact analytical) solution of the temperature field, applying the inverse integral transform (Laplace transform) of the Eq. (34), we get;

35 $$\begin{gathered} \vartheta_{1} (y,\varepsilon ) = \sum\limits_{l = 0}^{\infty } {\sum\limits_{m = 0}^{\infty } {\sum\limits_{n = 0}^{\infty } {\frac{{\left( y \right)^{ - l} \left( {\eta_{1} \Pr } \right)^{{\frac{l}{2} - m}} \left( {\Pr } \right)^{m}\left( \alpha \right)^{n} \Gamma \left( {\frac{l}{2} + 1} \right)\Gamma \left( {m + n} \right)}}{{l!m!n!\left( {1 - \alpha } \right)^{m + n} \Gamma \left( {\frac{l}{2} - m + 1} \right)\Gamma \left( m \right)}}} } } \cdot \frac{{\varepsilon^{n\alpha } }}{{\Gamma \left( {n\alpha + 1} \right)}}, \hfill \\ \vartheta_{2} (y,\varepsilon ) = ae^{ - t} *\sum\limits_{l = 0}^{\infty } {\sum\limits_{m = 0}^{\infty } {\sum\limits_{n = 0}^{\infty } {\frac{{\left( y \right)^{ - l} \left( {\eta_{1} \Pr } \right)^{{\frac{l}{2} - m}} \left( {\Pr } \right)^{m} \left( \alpha \right)^{n} \Gamma \left( {\frac{l}{2} + 1} \right)\Gamma \left( {m + n} \right)}}{{l!m!n!\left( {1 - \alpha } \right)^{m + n} \Gamma \left( {\frac{l}{2} - m + 1} \right)\Gamma \left( m \right)}}} } } \cdot \frac{{\varepsilon^{n\alpha } }}{{\Gamma \left( {n\alpha + 1} \right)}}, \hfill \\ \vartheta (y,\varepsilon ) = \vartheta_{1} (y,\varepsilon ) - \vartheta_{2} (y,\varepsilon ) \hfill \\ \end{gathered}$$

#### By Atangana Baleanu fractional derivative

After applying the integral transformation technique (Laplace transformation) to Eq. (12) with conditions (initial and boundary) given in Eq. (9), we have;

$$\frac{{\partial^{{^{2} }} \overline{\vartheta }(y,\varepsilon )}}{{\partial^{{^{2} }} y}} - \left( {\Pr \left( {\frac{{q^{{^{\alpha } }} }}{{(1 - \alpha )q^{{^{\alpha } }} + \alpha }} + \eta_{1} } \right)} \right)\overline{\vartheta }(y,q) = 0,$$

With the transformed conditions after taking Laplace transform;

$$\overline{\vartheta }(y,q) \to 0{\text{ as y}} \to \infty {\text{ and }}\overline{\vartheta }(0,q) = \frac{1}{q} - \frac{a}{q - b}.$$

The transformed solution obtained for the Temperature field is presented as:

36 $$\overline{\vartheta }(y,q) = \left( {\frac{1}{q} - \frac{a}{q + b}} \right)\exp \left( { - y\sqrt {\Pr \left( {\frac{{q^{{^{\alpha } }} }}{{(1 - \alpha )q^{{^{\alpha } }} + \alpha }} + \eta_{1} } \right)} } \right),$$

The equivalent series representation of the solution of the concentration field is represented as;

37 $$\overline{\vartheta }(y,q) = \left( {\frac{1}{q} - \frac{a}{q + b}} \right)\sum\limits_{l = 0}^{\infty } {\sum\limits_{m = 0}^{\infty } {\sum\limits_{n = 0}^{\infty } {\frac{{\left( y \right)^{ - l} \left( {\eta_{{_{1} }} \Pr } \right)^{{\frac{l}{2} - m}} \left( {\Pr } \right)^{m} \left( \alpha \right)^{n} \Gamma \left( {\frac{l}{2} + 1} \right)\Gamma \left( {m + n} \right)}}{{l!m!n!\left( {1 - \alpha } \right)^{m + n}  \Gamma \left( {\frac{l}{2} - m + 1} \right)\Gamma \left( m \right)}}} } } \cdot \frac{1}{{q^{n\alpha + 1} }},$$

For the purpose of obtaining the final result (exact analytical) solution of the temperature field, after taking the inverse integral transformation (inverse Laplace) of the Eq. (37), we get;

38 $$\begin{gathered} \vartheta_{1} (y,\varepsilon ) = \sum\limits_{l = 0}^{\infty } {\sum\limits_{m = 0}^{\infty } {\sum\limits_{n = 0}^{\infty } {\frac{{\left( y \right)^{ - l} \left( {\eta_{1} \Pr } \right)^{{\frac{l}{2} - m}} \left( {\Pr } \right)^{m} \left( \alpha \right)^{n} \Gamma \left( {\frac{l}{2} + 1} \right)\Gamma \left( {m + n} \right)}}{{l!m!n!\left( {1 - \alpha } \right)^{m + n} \Gamma \left( {\frac{l}{2} - m + 1} \right)\Gamma \left( m \right)}}} } } \cdot \frac{{\varepsilon^{n\alpha } }}{{\Gamma \left( {n\alpha + 1} \right)}}, \hfill \\ \vartheta_{2} (y,\varepsilon ) = ae^{ - \varepsilon } *\sum\limits_{l = 0}^{\infty } {\sum\limits_{m = 0}^{\infty } {\sum\limits_{n = 0}^{\infty } {\frac{{\left( y \right)^{ - l} \left( {\eta_{1} \Pr } \right)^{{\frac{l}{2} - m}} \left( {\Pr } \right)^{m} \left( \alpha \right)^{n} \Gamma \left( {\frac{l}{2} + 1} \right)\Gamma \left( {m + n} \right)}}{{l!m!n!\left( {1 - \alpha } \right)^{m + n}\Gamma \left( {\frac{l}{2} - m + 1} \right)\Gamma \left( m \right)}}} } } \cdot \frac{{\varepsilon^{n\alpha }}}{{\Gamma \left( {n\alpha + 1} \right)}}, \hfill \\ \vartheta (y,\varepsilon ) = \vartheta_{1} (y,\varepsilon ) - \vartheta_{2} (y,\varepsilon ) \hfill \\ \end{gathered}$$

#### By constant proportional-Caputo hybrid fractional derivative

After applying the integral transform technique to Eq. (11) with the corresponding conditions (IC’s and BC’s) given in Eq. (9), we have;

$$\frac{{\partial^{{^{2} }} \overline{\vartheta }(y,q)}}{{\partial^{{^{2} }} y}} - \left( {\Pr \left( {q^{{^{\alpha } }} \left[ {\frac{{k_{{_{1} }} (\alpha )}}{q} + k_{{_{0} }} (\alpha )} \right] + \eta_{1} } \right)} \right)\overline{\vartheta }(y,q) = 0,$$

With the transformed conditions after taking the Laplace transform;

$$\overline{\vartheta }(y,q) \to 0{\text{ as y}} \to \infty {\text{ and }}\overline{\vartheta }(0,q) = \frac{1}{q} - \frac{a}{q - b}.$$

The transformed solution obtained for the temperature field is interpreted as:

39 $$\overline{\vartheta }(y,q) = \left( {\frac{1}{q} - \frac{a}{q + b}} \right)\exp \left( { - y\sqrt {\Pr \left( {q^{{^{\alpha } }} \left[ {\frac{{k_{{_{1} }} (\alpha )}}{q} + k_{{_{0} }} (\alpha )} \right] + \eta_{1} } \right)} } \right),$$

The equivalent series representation of the solution of the concentration field is represented as;

40 $$\overline{\vartheta }(y,q) = \sum\limits_{l = 0}^{\infty } {\sum\limits_{m = 0}^{\infty } {\sum\limits_{n = 0}^{\infty } {\frac{{\left( y \right)^{ - l} \left( {\eta_{{_{1} }} \Pr } \right)^{{\frac{l}{2} - m}} \left( {\Pr } \right)^{m}\left( \alpha \right)^{n} \Gamma \left( {\frac{l}{2} + 1} \right)\Gamma \left( {m + n} \right)}}{{l!m!n!\left( {1 - \alpha } \right)^{m + n} \Gamma \left( {\frac{l}{2} - m + 1} \right)\Gamma \left( m \right)}}} } } \cdot \frac{1}{{q^{1 + n} }},$$

For the purpose of obtaining the final result of the temperature field, applying the inverse integral transformation (inverse Laplace) of the Eq. (40), we get;

41 $$\begin{gathered} \vartheta_{1} (y,\varepsilon ) = \sum\limits_{l = 0}^{\infty } {\sum\limits_{m = 0}^{\infty } {\sum\limits_{n = 0}^{\infty } {\frac{{\left( y \right)^{ - l} \left( {\eta_{1} \Pr } \right)^{{\frac{l}{2} - m}} \left( {\Pr } \right)^{m} \left( \alpha \right)^{n} \Gamma \left( {\frac{l}{2} + 1} \right)\Gamma \left( {m + n} \right)}}{{l!m!n!\left( {1 - \alpha } \right)^{m + n} \Gamma \left( {\frac{l}{2} - m + 1} \right)\Gamma \left( m \right)}}} } } \cdot \frac{{\varepsilon^{n\alpha } }}{{\Gamma \left( {n\alpha + 1} \right)}}, \hfill \\ \vartheta_{2} (y,\varepsilon ) = ae^{ - \varepsilon } *\sum\limits_{l = 0}^{\infty } {\sum\limits_{m = 0}^{\infty } {\sum\limits_{n = 0}^{\infty } {\frac{{\left( y \right)^{ - l} \left( {\eta_{1} \Pr } \right)^{{\frac{l}{2} - m}} \left( {\Pr } \right)^{m}\left( \alpha \right)^{n} \Gamma \left( {\frac{l}{2} + 1} \right)\Gamma \left( {m + n} \right)}}{{l!m!n!\left( {1 - \alpha } \right)^{m + n} \Gamma \left( {\frac{l}{2} - m + 1} \right)\Gamma \left( m \right)}}} } } \cdot \frac{{\varepsilon^{n\alpha } }}{{\Gamma \left( {n\alpha + 1} \right)}}, \hfill \\ \vartheta (y,\varepsilon ) = \vartheta_{1} (y,\varepsilon ) - \vartheta_{2} (y,\varepsilon ) \hfill \\ \end{gathered}$$

### Exact solution of fluid velocity

#### By Caputo Fabrizio fractional derivative

Solution/final result of the velocity equation described in Eq. (13) with after the Laplace transformation, we obtain the following form of the velocity equation;

$$\begin{gathered} \left[ {\frac{q}{(1 - \alpha )q + \alpha }} \right]\overline{w}(y,q) = \left( {1 + \alpha_{{_{2} }} \left[ {\frac{q}{(1 - \alpha )q + \alpha }} \right]} \right)\frac{{\partial \overline{w}^{{^{2} }} (y,q)}}{{\partial y^{{^{2} }} }} + G_{r} \overline{\vartheta }\left( {y,q} \right) \hfill \\ { - }\left( {M + \frac{1}{K}\left( {1 + \alpha_{{_{2} }} \left[ {\frac{q}{(1 - \alpha )q + \alpha }} \right]} \right)} \right)\overline{w}(y,q) + G_{m} \overline{C}\left( {y,q} \right) \, \hfill \\ \end{gathered}$$

After some rearrangements the transformed velocity equation can be presented as:

$$\begin{gathered} \hfill \\ \frac{{\partial \overline{w}^{{^{2} }} (y,q)}}{{\partial y^{{^{2} }} }} - \left( {\frac{{b_{{_{1} }} q + a_{{_{1} }} }}{{b_{{_{2} }} q + \alpha }}} \right)\overline{w}(y,q) = - \left( {\frac{{b_{{_{3} }} q + \alpha }}{{b_{{_{2} }} q + \alpha }}} \right)\left( {G_{r} \overline{\vartheta }\left( {y,q} \right) + G_{m} \overline{C}\left( {y,q} \right)} \right) \hfill \\ \end{gathered}$$

where $$C_{1} = M + \frac{1}{K},{\text{ C}}_{{_{2} }} = \frac{{\alpha_{{_{2} }} }}{K}{\text{ and d}}_{{_{1} }} = 1 + C_{{_{2} }} .$$

By using the corresponding IC’s and BC’s condition $\overline{w}(y,q) \to 0{\text{ as y}} \to \infty {\text{ and }}\overline{w}(0,q) = \frac{1}{q - \omega }$, we obtained the analytical solution of velocity equation as;

42 $$\begin{aligned} \overline{w}(y,q) & = \frac{1}{q - \omega } \times e^{{\sqrt {\frac{{b_{1} q + a_{1} }}{{b_{2} q + \alpha }}} }} + \frac{{Gr\left( {\frac{1}{q} - \frac{a}{q + b}} \right)\left( {b_{3} q + \alpha } \right)^{2} }}{{\Pr q + \Pr \eta_{1} \left( {b_{3} q + \alpha } \right)\left( {b_{2} q + \alpha } \right) - \left( {b_{1} q + a_{1} } \right)\left( {b_{3} q + \alpha } \right)}} \times \left[ {e^{{\sqrt {\frac{{b_{1} q + a_{1} }}{{b_{2} q + \alpha }}} }} - e^{{\sqrt {\frac{\Pr \cdot q}{{\left( {b_{3} q + \alpha } \right)}} + \Pr \cdot \eta_{{_{1} }} } }} } \right] \\ + \frac{{Gm\left( {\frac{1}{q - \omega }} \right)\left( {b_{3} q + \alpha } \right)^{2} }}{{Sc \cdot q + Sc \cdot \eta_{2} \left( {b_{3} q + \alpha } \right)\left( {b_{2} q + \alpha } \right) - \left( {b_{1} q + a_{1} } \right)\left( {b_{3} q + \alpha } \right)}} \times \left[ {e^{{\sqrt {\frac{{b_{1} q + a_{1} }}{{b_{2} q + \alpha }}} }} - e^{{\sqrt {\frac{Sc \cdot q}{{b_{3} q + \alpha }} + Sc \cdot \eta_{2} } }} } \right] \\ \end{aligned}$$

The simplified form of the series representation of the velocity equation is represented as;

43 $$\overline{w}(y,q) = \overline{A}_{3} (y,q) + Gr\overline{A}_{4} (y,q)\left\{ {\overline{A}_{3} (y,q) - a\overline{A}_{5} (y,q) - \overline{\vartheta }(y,q)} \right\} + Gm\overline{A}_{6} (y,q)\left\{ {\overline{A}_{3} (y,q) - \overline{C}(y,q)} \right\},$$

where

$$\begin{gathered} \overline{A}_{3} (y,q) = \overline{A}_{1} (y,q)\overline{A}_{2} (y,q) \hfill \\ = \sum\limits_{v = 0}^{\infty } {\sum\limits_{p = 0}^{\infty } {\sum\limits_{h = 0}^{\infty } {\frac{{\left( { - 1} \right)^{h} \left( { - y} \right)^{\nu } \left( {a_{1} } \right)^{p} \left( {b_{1} } \right)^{{\frac{\nu }{2} - p}} \left( \alpha \right)^{h} \Gamma \left( {\frac{\nu }{2} + 1} \right)\Gamma \left( {p + h} \right)}}{{\nu !p!h!\left( {b_{2} } \right)^{p + h} \Gamma \left( {\frac{\nu }{2} - p + 1} \right)\Gamma \left( h \right)}}\frac{1}{{q^{\alpha p + \alpha h} }}} } } , \hfill \\ \overline{A}_{4} (y,q) = \left( {b_{3} q^{\alpha } + \alpha } \right)^{2} \frac{1}{{\left( {\Pr b_{2} - b_{1} b_{3} } \right)q^{\alpha 2} - \left( {a_{1} b_{3} + b_{1} \alpha - \Pr \alpha } \right)q^{\alpha } - a_{1} \alpha }} \hfill \\ = b_{3}^{2} \sum\limits_{n = 0}^{\infty } {\frac{{\delta^{n} }}{{\left( {\Pr b_{2} - b_{1} b_{3} } \right)^{n + 1} }}\frac{{(q)^{ - n + 1} }}{{\left( {q^{\alpha } - \frac{{\left( {a_{1} b_{3} + b_{1} \alpha - \Pr \alpha } \right)}}{{\Pr b_{2} - b_{1} b_{3} }}} \right)^{n + 1} }}} + 2b_{3} \alpha \sum\limits_{n = 0}^{\infty } {\frac{{\delta^{n} }}{{\left( {\Pr b_{2} - b_{1} b_{3} } \right)^{n + 1} }}\frac{{(q)^{ - n + 1} }}{{\left( {q^{\alpha } - \frac{{\left( {a_{1} b_{3} + b_{1} \alpha - \Pr \alpha } \right)}}{{\Pr b_{2} - b_{1} b_{3} }}} \right)^{n + 1} }}} \hfill \\ + \alpha^{2} \sum\limits_{n = 0}^{\infty } {\frac{{\delta^{n} }}{{\left( {\Pr b_{2} - b_{1} b_{3} } \right)^{n + 1} }}\frac{{(q)^{ - n + 1} }}{{\left( {q^{\alpha } - \frac{{\left( {a_{1} b_{3} + b_{1} \alpha - \Pr \alpha } \right)}}{{\Pr b_{2} - b_{1} b_{3} }}} \right)^{n + 1} }}} , \hfill \\ \overline{A}_{5} (y,q) = \frac{1}{q + b}\left( {\sum\limits_{v = 0}^{\infty } {\sum\limits_{p = 0}^{\infty } {\sum\limits_{h = 0}^{\infty } {\frac{{\left( { - 1} \right)^{h} \left( { - y} \right)^{\nu } \left( {a_{1} } \right)^{p} \left( {b_{1} } \right)^{{\frac{\nu }{2} - p}} \left( \alpha \right)^{h} \Gamma \left( {\frac{\nu }{2} + 1} \right)\Gamma \left( {p + h} \right)}}{{\nu !p!h!\left( {b_{2} } \right)^{p + h} \Gamma \left( {\frac{\nu }{2} - p + 1} \right)\Gamma \left( h \right)}}\frac{1}{{q^{\alpha p + \alpha h} }}} } } } \right), \hfill \\ \overline{A}_{6} (y,q) = \left( {b_{3} q^{\alpha } + \alpha } \right)^{2} \frac{1}{{\left( {Scb_{2} - b_{1} b_{3} } \right)q^{\alpha 2} - \left( {a_{1} b_{3} + b_{1} \alpha - Sc\alpha } \right)q^{\alpha } - a_{1} \alpha }} \hfill \\ = b_{3}^{2} \sum\limits_{n = 0}^{\infty } {\frac{{\delta^{n} }}{{\left( {Scb_{2} - b_{1} b_{3} } \right)^{n + 1} }}\frac{{(q)^{ - n + 1} }}{{\left( {q^{\alpha } - \frac{{\left( {a_{1} b_{3} + b_{1} \alpha - Sc\alpha } \right)}}{{Scb_{2} - b_{1} b_{3} }}} \right)^{n + 1} }}} + 2b_{3} \alpha \sum\limits_{n = 0}^{\infty } {\frac{{\delta^{n} }}{{\left( {Scb_{2} - b_{1} b_{3} } \right)^{n + 1} }}\frac{{(q)^{ - n + 1} }}{{\left( {q^{\alpha } - \frac{{\left( {a_{1} b_{3} + b_{1} \alpha - Sc\alpha } \right)}}{{Scb_{2} - b_{1} b_{3} }}} \right)^{n + 1} }}} \hfill \\ + \alpha^{2} \sum\limits_{n = 0}^{\infty } {\frac{{\delta^{n} }}{{\left( {Scb_{2} - b_{1} b_{3} } \right)^{n + 1} }}\frac{{(q)^{ - n + 1} }}{{\left( {q^{\alpha } - \frac{{\left( {a_{1} b_{3} + b_{1} \alpha - \Pr \alpha } \right)}}{{Scb_{2} - b_{1} b_{3} }}} \right)^{n + 1} }}} . \hfill \\ \end{gathered}$$

After taking the inverse integral transform (Laplace inverse) of the velocity field solution;

44 $$w(y,\varepsilon ) = A_{3} (y,\varepsilon ) + GrA_{4} (y,\varepsilon )\left\{ {A_{3} (y,\varepsilon ) - aA_{5} (y,\varepsilon ) - \vartheta (y,\varepsilon )} \right\} + GrA_{6} (y,\varepsilon )\left\{ {A_{3} (y,\varepsilon ) - C(y,\varepsilon )} \right\},$$

where

$$\begin{gathered} A_{3} (y,\varepsilon ) = A_{1} (y,\varepsilon )A_{2} (y,\varepsilon ) \hfill \\ { = }\sum\limits_{v = 0}^{\infty } {\sum\limits_{p = 0}^{\infty } {\sum\limits_{h = 0}^{\infty } {\frac{{\left( { - 1} \right)^{h} \left( { - y} \right)^{\nu } \left( {a_{1} } \right)^{p} \left( {b_{1} } \right)^{{\frac{\nu }{2} - p}} \left( \alpha \right)^{h} \Gamma \left( {\frac{\nu }{2} + 1} \right)\Gamma \left( {p + h} \right)}}{{\nu !p!h!\left( {b_{2} } \right)^{p + h} \Gamma \left( {\frac{\nu }{2} - p + 1} \right)\Gamma \left( h \right)}}\frac{{\varepsilon^{\alpha p + \alpha h - 1} }}{{\Gamma \left( {\alpha p + \alpha h} \right)}}} } } , \hfill \\ A_{4} (y,\varepsilon ) = \sum\limits_{n = 0}^{\infty } {\frac{{\delta^{n} }}{{\left( \beta \right)^{n + 1} }}} \left( \begin{gathered} b_{3}^{2} G_{1, - \alpha n + 1,\alpha n + 1} \left( {\frac{{\left( {a_{1} b_{3} + b_{1} \alpha - \Pr \alpha } \right)}}{{\Pr b_{2} - b_{1} b_{3} }},\varepsilon } \right) + 2b_{3} \alpha G_{1, - \alpha n + 1,\alpha n + 1} \left( {\frac{{\left( {a_{1} b_{3} + b_{1} \alpha - \Pr \alpha } \right)}}{{\Pr b_{2} - b_{1} b_{3} }},\varepsilon } \right) \hfill \\ + \alpha^{2} G_{1, - \alpha n + 1,\alpha n + 1} \left( {\frac{{\left( {a_{1} b_{3} + b_{1} \alpha - \Pr \alpha } \right)}}{{\Pr b_{2} - b_{1} b_{3} }},\varepsilon } \right) \hfill \\ \end{gathered} \right) \hfill \\ \, \hfill \\ A_{5} (y,\varepsilon ) = e^{ - b\varepsilon } *\left( {\sum\limits_{v = 0}^{\infty } {\sum\limits_{p = 0}^{\infty } {\sum\limits_{h = 0}^{\infty } {\frac{{\left( { - 1} \right)^{h} \left( { - y} \right)^{\nu } \left( {a_{1} } \right)^{p} \left( {b_{1} } \right)^{{\frac{\nu }{2} - p}} \left( \alpha \right)^{h} \Gamma \left( {\frac{\nu }{2} + 1} \right)\Gamma \left( {p + h} \right)}}{{\nu !p!h!\left( {b_{2} } \right)^{p + h} \Gamma \left( {\frac{\nu }{2} - p + 1} \right)\Gamma \left( h \right)}}\frac{{\varepsilon^{\alpha p + \alpha h} }}{{\Gamma \left( {\alpha h + \alpha p + 1} \right)}}} } } } \right), \hfill \\ A_{6} (y,\varepsilon ) = \sum\limits_{n = 0}^{\infty } {\frac{{\delta^{n} }}{{\left( \beta \right)^{n + 1} }}} \left( \begin{gathered} b_{3}^{2} G_{1, - \alpha n + 1,\alpha n + 1} \left( {\frac{{\left( {a_{1} b_{3} + b_{1} \alpha - Sc\alpha } \right)}}{{Scb_{2} - b_{1} b_{3} }},\varepsilon } \right) + 2b_{3} \alpha G_{1, - \alpha n + 1,\alpha n + 1} \left( {\frac{{\left( {a_{1} b_{3} + b_{1} \alpha - Sc\alpha } \right)}}{{Scb_{2} - b_{1} b_{3} }},\varepsilon } \right) \hfill \\ + \alpha^{2} G_{1, - \alpha n + 1,\alpha n + 1} \left( {\frac{{\left( {a_{1} b_{3} + b_{1} \alpha - Sc\alpha } \right)}}{{Scb_{2} - b_{1} b_{3} }},\varepsilon } \right) \hfill \\ \end{gathered} \right) \hfill \\ \end{gathered}$$

#### By Atangana Baleanu fractional derivative

Solution of the velocity equation described in Eq. (16) after the Laplace transformation, we achieve the following form of the velocity equation;

$$\begin{aligned} \left[ {\frac{{q^{{^{\alpha } }} }}{{(1 - \alpha )q^{{^{\alpha } }} + \alpha }}} \right]\overline{w}(y,q) & = \left( {1 + \alpha_{{_{2} }} \left[ {\frac{{q^{{^{\alpha } }} }}{{(1 - \alpha )q^{{^{\alpha } }} + \alpha }}} \right]} \right)\frac{{\partial \overline{w}^{{^{2} }} (y,q)}}{{\partial y^{{^{2} }} }} + G_{r} \overline{\vartheta }\left( {y,q} \right) \\ & \quad - \left( {M + \frac{1}{K}\left( {1 + \alpha_{{_{2} }} \left[ {\frac{{q^{{^{\alpha } }} }}{{(1 - \alpha )q^{{^{\alpha } }} + \alpha }}} \right]} \right)} \right)\overline{w}(y,q) + G_{m} \overline{C}\left( {y,q} \right){, } \\ \end{aligned}$$

After some rearrangements the transformed velocity equation can be described as:

$$\begin{gathered} \hfill \\ \frac{{\partial \overline{w}^{{^{2} }} (y,q)}}{{\partial y^{{^{2} }} }} - \left( {\frac{{b_{{_{1} }} q^{{^{\alpha } }} + a_{{_{1} }} }}{{b_{{_{2} }} q^{{^{\alpha } }} + \alpha }}} \right)\overline{w}(y,q) = - \left( {\frac{{b_{{_{3} }} q^{{^{\alpha } }} + \alpha }}{{b_{{_{2} }} q^{{^{\alpha } }} + \alpha }}} \right)\left( {G_{r} \overline{\vartheta }\left( {y,q} \right) + G_{m} \overline{C}\left( {y,q} \right)} \right) \hfill \\ \end{gathered}$$

where $$C_{1} = M + \frac{1}{K},{\text{ C}}_{{_{2} }} = \frac{{\alpha_{{_{2} }} }}{K}{\text{ and d}}_{{_{1} }} = 1 + C_{{_{2} }} .$$

By using the related IC’s (initial) and BC’s (boundary) condition $\overline{w}(y,q) \to 0 \, as \, y \to \infty {\text{ and }}\overline{w}(0,q) = \frac{1}{q - \omega }$, we obtained the solution of velocity equation as;

45 $$\begin{gathered} \overline{w}(y,q) = \frac{1}{q - \omega } \times e^{{\sqrt {\frac{{b_{1} q^{{^{\alpha } }} + a_{1} }}{{b_{2} q^{\alpha } + \alpha }}} }} + \frac{{Gr\left( {\frac{1}{q} - \frac{a}{q + b}} \right)\left( {b_{3} q^{\alpha } + \alpha } \right)^{{^{2} }} }}{{\Pr s + \Pr \eta_{1} \left( {b_{3} q^{\alpha } + \alpha } \right)\left( {b_{2} q^{\alpha } + \alpha } \right) - \left( {b_{1} q^{\alpha } + a_{1} } \right)\left( {b_{3} q^{\alpha } + \alpha } \right)}} \times \left[ {e^{{\sqrt {\frac{{b_{1} q^{{^{\alpha } }} + a_{1} }}{{b_{2} q^{\alpha } + \alpha }}} }} - e^{{\sqrt {\frac{{\Pr \cdot q^{\alpha } }}{{\left( {b_{3} q^{\alpha } + \alpha } \right)}} + \Pr \cdot \eta_{1} } }} } \right] \hfill \\ { + }\frac{{Gm\left( {\frac{1}{q - \omega }} \right)\left( {b_{{_{3} }} q^{\alpha } + \alpha } \right)^{{^{2} }} }}{{Sc \cdot q^{\alpha } + Sc \cdot \eta_{2} \left( {b_{3} q^{\alpha } + \alpha } \right)\left( {b_{2} q^{\alpha } + \alpha } \right) - \left( {b_{1} q^{{^{\alpha } }} + a_{1} } \right)\left( {b_{3} q^{\alpha } + \alpha } \right)}} \times \left[ {e^{{\sqrt {\frac{{b_{1} q^{\alpha } + a_{1} }}{{b_{2} q^{\alpha } + \alpha }}} }} - e^{{\sqrt {\frac{{Sc \cdot q^{\alpha } }}{{b_{3} q^{\alpha } + \alpha }} + Sc \cdot \eta_{2} } }} } \right] \hfill \\ \end{gathered}$$

The simplified form of the series representation of the velocity equation solution is represented as;

$$\begin{gathered} \overline{w}(y,q) = \overline{A}_{3} (y,q) + Gr\overline{A}_{4} (y,q)\left\{ {\overline{A}_{3} (y,q) - a\overline{A}_{5} (y,q) - \overline{\vartheta }(y,q)} \right\} + Gm\overline{A}_{6} (y,q)\left\{ {\overline{A}_{3} (y,q) - \overline{C}(y,q)} \right\}, \hfill \\ {\text{where}} \hfill \\ \overline{A}_{3} (y,q) = \overline{A}_{1} (y,q)\overline{A}_{2} (y,q) \hfill \\ \, = \sum\limits_{v = 0}^{\infty } {\sum\limits_{p = 0}^{\infty } {\sum\limits_{h = 0}^{\infty } {\frac{{\left( { - 1} \right)^{h} \left( { - y} \right)^{\nu } \left( {a_{1} } \right)^{p} \left( {b_{1} } \right)^{{\frac{\nu }{2} - p}} \left( \alpha \right)^{h} \Gamma \left( {\frac{\nu }{2} + 1} \right)\Gamma \left( {p + h} \right)}}{{\nu !p!h!\left( {b_{2} } \right)^{p + h} \Gamma \left( {\frac{\nu }{2} - p + 1} \right)\Gamma \left( h \right)}}\frac{1}{{q^{\alpha p + \alpha h} }}} } } , \hfill \\ \overline{A}_{4} (y,q) = \left( {b_{3} q^{\alpha } + \alpha } \right)^{2} \frac{1}{{\left( {\Pr b_{2} - b_{1} b_{3} } \right)q^{\alpha 2} - \left( {a_{1} b_{3} + b_{1} \alpha - \Pr \alpha } \right)q^{\alpha } - a_{1} \alpha }} \hfill \\ \, = b_{3}^{2} \sum\limits_{n = 0}^{\infty } {\frac{{\delta^{n} }}{{\left( {\Pr b_{2} - b_{1} b_{3} } \right)^{n + 1} }}\frac{{(q)^{ - n + 1} }}{{\left( {q^{\alpha } - \frac{{\left( {a_{1} b_{3} + b_{1} \alpha - \Pr \alpha } \right)}}{{\Pr b_{2} - b_{1} b_{3} }}} \right)^{n + 1} }}} + 2b_{3} \alpha \sum\limits_{n = 0}^{\infty } {\frac{{\delta^{n} }}{{\left( {\Pr b_{2} - b_{1} b_{3} } \right)^{n + 1} }}\frac{{(q)^{ - n + 1} }}{{\left( {q^{\alpha } - \frac{{\left( {a_{1} b_{3} + b_{1} \alpha - \Pr \alpha } \right)}}{{\Pr b_{2} - b_{1} b_{3} }}} \right)^{n + 1} }}} \hfill \\ \, + \alpha^{2} \sum\limits_{n = 0}^{\infty } {\frac{{\delta^{n} }}{{\left( {\Pr b_{2} - b_{1} b_{3} } \right)^{n + 1} }}\frac{{(q)^{ - n + 1} }}{{\left( {q^{\alpha } - \frac{{\left( {a_{1} b_{3} + b_{1} \alpha - \Pr \alpha } \right)}}{{\Pr b_{2} - b_{1} b_{3} }}} \right)^{n + 1} }}} , \hfill \\ \overline{A}_{5} (y,q) = \frac{1}{s + b}\left( {\sum\limits_{v = 0}^{\infty } {\sum\limits_{p = 0}^{\infty } {\sum\limits_{h = 0}^{\infty } {\frac{{\left( { - 1} \right)^{h} \left( { - y} \right)^{\nu } \left( {a_{1} } \right)^{p} \left( {b_{1} } \right)^{{\frac{\nu }{2} - p}} \left( \alpha \right)^{h} \Gamma \left( {\frac{\nu }{2} + 1} \right)\Gamma \left( {p + h} \right)}}{{\nu !p!h!\left( {b_{2} } \right)^{p + h} \Gamma \left( {\frac{\nu }{2} - p + 1} \right)\Gamma \left( h \right)}}\frac{1}{{q^{\alpha p + \alpha h} }}} } } } \right), \hfill \\ \overline{A}_{6} (y,q) = \left( {b_{3} q^{\alpha } + \alpha } \right)^{2} \frac{1}{{\left( {Scb_{2} - b_{1} b_{3} } \right)q^{\alpha 2} - \left( {a_{1} b_{3} + b_{1} \alpha - Sc\alpha } \right)q^{\alpha } - a_{1} \alpha }} \hfill \\ \, = b_{3}^{2} \sum\limits_{n = 0}^{\infty } {\frac{{\delta^{n} }}{{\left( {Scb_{2} - b_{1} b_{3} } \right)^{n + 1} }}\frac{{(q)^{ - n + 1} }}{{\left( {q^{\alpha } - \frac{{\left( {a_{1} b_{3} + b_{1} \alpha - Sc\alpha } \right)}}{{Scb_{2} - b_{1} b_{3} }}} \right)^{n + 1} }}} + 2b_{3} \alpha \sum\limits_{n = 0}^{\infty } {\frac{{\delta^{n} }}{{\left( {Scb_{2} - b_{1} b_{3} } \right)^{n + 1} }}\frac{{(q)^{ - n + 1} }}{{\left( {q^{\alpha } - \frac{{\left( {a_{1} b_{3} + b_{1} \alpha - Sc\alpha } \right)}}{{Scb_{2} - b_{1} b_{3} }}} \right)^{n + 1} }}} \hfill \\ \, + \alpha^{2} \sum\limits_{n = 0}^{\infty } {\frac{{\delta^{n} }}{{\left( {Scb_{2} - b_{1} b_{3} } \right)^{n + 1} }}\frac{{(q)^{ - n + 1} }}{{\left( {q^{\alpha } - \frac{{\left( {a_{1} b_{3} + b_{1} \alpha - \Pr \alpha } \right)}}{{Scb_{2} - b_{1} b_{3} }}} \right)^{n + 1} }}} . \hfill \\ \end{gathered}$$

After the Laplace inverse transformation of the solution of velocity field;

$$\begin{gathered} w(y,\varepsilon ) = A_{3} (y,\varepsilon ) + GrA_{4} (y,\varepsilon )\left\{ {A_{3} (y,\varepsilon ) - aA_{5} (y,\varepsilon ) - \vartheta (y,\varepsilon )} \right\} + GrA_{6} (y,\varepsilon )\left\{ {A_{3} (y,\varepsilon ) - C(y,\varepsilon )} \right\}, \hfill \\ {\text{where}} \hfill \\ A_{3} (y,\varepsilon ) = A_{1} (y,\varepsilon )A_{2} (y,\varepsilon ) \hfill \\ { = }\sum\limits_{v = 0}^{\infty } {\sum\limits_{p = 0}^{\infty } {\sum\limits_{h = 0}^{\infty } {\frac{{\left( { - 1} \right)^{h} \left( { - y} \right)^{\nu } \left( {a_{1} } \right)^{p} \left( {b_{1} } \right)^{{\frac{\nu }{2} - p}} \left( \alpha \right)^{h} \Gamma \left( {\frac{\nu }{2} + 1} \right)\Gamma \left( {p + h} \right)}}{{\nu !p!h!\left( {b_{2} } \right)^{p + h} \Gamma \left( {\frac{\nu }{2} - p + 1} \right)\Gamma \left( h \right)}}\frac{{\varepsilon^{\alpha p + \alpha h - 1} }}{{\Gamma \left( {\alpha p + \alpha h} \right)}}} } } , \hfill \\ A_{4} (y,\varepsilon ) = \sum\limits_{n = 0}^{\infty } {\frac{{\delta^{n} }}{{\left( \beta \right)^{n + 1} }}} \left( \begin{gathered} b_{3}^{2} G_{1, - \alpha n + 1,\alpha n + 1} \left( {\frac{{\left( {a_{1} b_{3} + b_{1} \alpha - \Pr \alpha } \right)}}{{\Pr b_{2} - b_{1} b_{3} }},\varepsilon } \right) + 2b_{3} \alpha G_{1, - \alpha n + 1,\alpha n + 1} \left( {\frac{{\left( {a_{1} b_{3} + b_{1} \alpha - \Pr \alpha } \right)}}{{\Pr b_{2} - b_{1} b_{3} }},\varepsilon } \right) \hfill \\ + \alpha^{2} G_{1, - \alpha n + 1,\alpha n + 1} \left( {\frac{{\left( {a_{1} b_{3} + b_{1} \alpha - \Pr \alpha } \right)}}{{\Pr b_{2} - b_{1} b_{3} }},\varepsilon } \right) \hfill \\ \end{gathered} \right) \hfill \\ \, \hfill \\ A_{5} (y,\varepsilon ) = e^{ - b\varepsilon } *\left( {\sum\limits_{v = 0}^{\infty } {\sum\limits_{p = 0}^{\infty } {\sum\limits_{h = 0}^{\infty } {\frac{{\left( { - 1} \right)^{h} \left( { - y} \right)^{\nu } \left( {a_{1} } \right)^{p} \left( {b_{1} } \right)^{{\frac{\nu }{2} - p}} \left( \alpha \right)^{h} \Gamma \left( {\frac{\nu }{2} + 1} \right)\Gamma \left( {p + h} \right)}}{{\nu !p!h!\left( {b_{2} } \right)^{p + h} \Gamma \left( {\frac{\nu }{2} - p + 1} \right)\Gamma \left( h \right)}}\frac{{\varepsilon^{\alpha p + \alpha h} }}{{\Gamma \left( {\alpha h + \alpha p + 1} \right)}}} } } } \right), \hfill \\ A_{6} (y,\varepsilon ) = \sum\limits_{n = 0}^{\infty } {\frac{{\delta^{n} }}{{\left( \beta \right)^{n + 1} }}} \left( \begin{gathered} b_{3}^{2} G_{1, - \alpha n + 1,\alpha n + 1} \left( {\frac{{\left( {a_{1} b_{3} + b_{1} \alpha - Sc\alpha } \right)}}{{Scb_{2} - b_{1} b_{3} }},\varepsilon } \right) + 2b_{3} \alpha G_{1, - \alpha n + 1,\alpha n + 1} \left( {\frac{{\left( {a_{1} b_{3} + b_{1} \alpha - Sc\alpha } \right)}}{{Scb_{2} - b_{1} b_{3} }},\varepsilon } \right) \hfill \\ + \alpha^{2} G_{1, - \alpha n + 1,\alpha n + 1} \left( {\frac{{\left( {a_{1} b_{3} + b_{1} \alpha - Sc\alpha } \right)}}{{Scb_{2} - b_{1} b_{3} }},\varepsilon } \right) \hfill \\ \end{gathered} \right) \hfill \\ \end{gathered}$$

#### By constant proportional-Caputo hybrid fractional derivative

Solution of the velocity equation represented in Eq. (10) by CPC fractional operator with the application of integral transform (Laplace transformation) technique, we obtain the following form of the velocity equation;

$$\begin{aligned} \left[ {\left( {\frac{{k_{{_{1} }} (\alpha )}}{q} - k_{{_{0} }} (\alpha )} \right)q^{{^{\alpha } }} } \right]\overline{w}(y,q) & = \left( {1 + \alpha_{{_{2} }} \left( {\frac{{k_{{_{1} }} (\alpha )}}{q} - k_{{_{0} }} (\alpha )} \right)q^{{^{\alpha } }} } \right)\frac{{\partial \overline{w}^{{^{2} }} (y,q)}}{{\partial y^{{^{2} }} }} + G_{r} \overline{\vartheta }\left( {y,q} \right) \\ & - \left( {M + \frac{1}{K}\left( {1 + \alpha_{{_{2} }} \left( {\frac{{k_{{_{1} }} (\alpha )}}{q} - k_{{_{0} }} (\alpha )} \right)q^{{^{\alpha } }} } \right)} \right)\overline{w}(y,q) + G_{m} \overline{C}\left( {y,q} \right) \, \\ \end{aligned}$$

Rearranging the transformed velocity equation can be expressed as:

$$\begin{gathered} \hfill \\ \frac{{\partial \overline{w}^{{^{2} }} (y,q)}}{{\partial y^{{^{2} }} }} - \left( {\frac{{C_{{_{1} }} + d_{{_{1} }} w(q)}}{{1 + \alpha_{{_{2} }} w(q)}}} \right)\overline{w}(y,q) = - \left( {\frac{1}{{1 + \alpha_{{_{2} }} w(q)}}} \right)\left( {G_{r} \overline{\vartheta }\left( {y,q} \right) + G_{m} \overline{C}\left( {y,q} \right)} \right) \hfill \\ \end{gathered}$$

where $$C_{1} = M + \frac{1}{K},{\text{ C}}_{{_{2} }} = \frac{{\alpha_{{_{2} }} }}{K}{\text{ and d}}_{{_{1} }} = 1 + C_{{_{2} }} .$$

By making use of corresponding/related IC’s (initial) and BC’s (boundary) conditions $\overline{w}(y,q) \to 0{\text{ as y}} \to \infty {\text{ and }}\overline{w}(0,q) = \frac{1}{q - \omega }$, we obtained the solution of velocity equation as;

46 $$\begin{gathered} \overline{w}(y,q) = \frac{1}{q - \omega } \times e^{{\sqrt {\frac{{C_{1} + d_{1} w(q)}}{{1 + \alpha_{{_{2} }} w(q)}}} }} + \frac{{Gr\left( {\frac{1}{q} - \frac{a}{q + b}} \right)}}{{\Pr \left( {w(q) + \eta_{{_{1} }} )} \right)\left( {1 + \alpha_{{_{2} }} w(q)} \right) - \left( {C_{1} + d_{1} w(q)} \right)}} \times \left[ {e^{{\sqrt {\frac{{C_{1} + d_{1} w(q)}}{{1 + \alpha_{{_{2} }} w(q)}}} }} - e^{{\sqrt {\Pr \left( {w(q) + \eta_{{_{1} }} )} \right)} }} } \right] \hfill \\ { + }\frac{{Gm\left( {\frac{1}{q - \omega }} \right)}}{{Sc\left( {w(q) + \eta_{{_{2} }} )} \right)\left( {1 + \alpha_{{_{2} }} w(q)} \right) - \left( {C_{1} + d_{1} w(q)} \right)}} \times \left[ {e^{{\sqrt {\frac{{C_{1} + d_{1} w(q)}}{{1 + \alpha_{{_{2} }} w(q)}}} }} - e^{{\sqrt {Sc\left( {w(q) + \eta_{{_{{_{2} }} }} )} \right)} }} } \right] \hfill \\ \end{gathered}$$

47 $$\begin{gathered} \overline{w}(y,q) = \overline{A}_{3} (y,q) + Gr\overline{A}_{4} (y,q)\left\{ {\overline{A}_{3} (y,q) - a\overline{A}_{5} (y,q) - \overline{\vartheta }(y,q)} \right\} + Gm\overline{A}_{6} (y,q)\left\{ {\overline{A}_{3} (y,q) - \overline{C}(y,q)} \right\} \hfill \\ w(y,\varepsilon ) = L^{ - 1} \left\{ \begin{gathered} \frac{1}{q - \omega } \times e^{{\sqrt {\frac{{C_{1} + d_{1} w(q)}}{{1 + \alpha_{2} w(q)}}} }} + \frac{{Gr\left( {\frac{1}{q} - \frac{a}{q + b}} \right)}}{{\Pr \left( {w(q) + \eta_{1} )} \right)\left( {1 + \alpha_{2} w(q)} \right) - \left( {C_{1} + d_{1} w(q)} \right)}} \times \left[ {e^{{\sqrt {\frac{{C_{1} + d_{1} w(q)}}{{1 + \alpha_{2} w(q)}}} }} - e^{{\sqrt {\Pr \left( {w(q) + \eta_{1} )} \right)} }} } \right] \hfill \\ \, + \frac{{Gm\left( {\frac{1}{q - \omega }} \right)}}{{Sc\left( {w(q) + \eta_{2} )} \right)\left( {1 + \alpha_{2} w(q)} \right) - \left( {C_{1} + d_{1} w(q)} \right)}} \times \left[ {e^{{\sqrt {\frac{{C_{1} + d_{1} w(q)}}{{1 + \alpha_{2} w(q)}}} }} - e^{{\sqrt {Sc\left( {w(q) + \eta_{2} )} \right)} }} } \right]\, \, \hfill \\ \end{gathered} \right\} \hfill \\ \end{gathered}$$

After inverse Laplace transformation of the solution of velocity field we get;

$$\begin{gathered} w(y,\varepsilon ) = A_{3} (y,\varepsilon ) + GrA_{4} (y,\varepsilon )\left\{ {A_{3} (y,\varepsilon ) - aA_{5} (y,\varepsilon ) - \vartheta (y,\varepsilon )} \right\} + GmA_{6} (y,\varepsilon )\left\{ {A_{3} (y,\varepsilon ) - C(y,\varepsilon )} \right\} \hfill \\ \hfill \\ \end{gathered}$$

$G_{a,b,c} (c,d)$ is called G-function and

$$\frac{{q^{a} }}{{\left( {q^{b} - c} \right)^{d} }} = L\left\{ {G_{a,b,c} (c,d)} \right\}$$

## Results and discussion

The generalized Second-Grade fluid flow described by three fractional derivatives i.e. CF, ABC and CPC fractional derivative is analyzed in this article. The heat mass transfer is observed during the flow past a vertical plate that is accelerated exponentially. The effects of the heat generation and exponential heating in the heat boundary layer and chemical reaction at the concentration boundary layer are also analyzed in this article. The fluid is flowing with exponentially variable velocity in a porous medium under the effects of the Magneto hydro dynamics. For the better understanding of the rheological properties of the Second Grade fluid we used the CF, ABC and CPC to interpret the memory effects. The exact solution/final result of the scheme is obtained in the form of G-functions and Mittag Leffler functions. For the physical significance of flow parameters, different parameters are graphed. From this analysis it is deduced that the CPC fractional operator is the most suitable operator to describe the memory effects.

**Figure 1:**
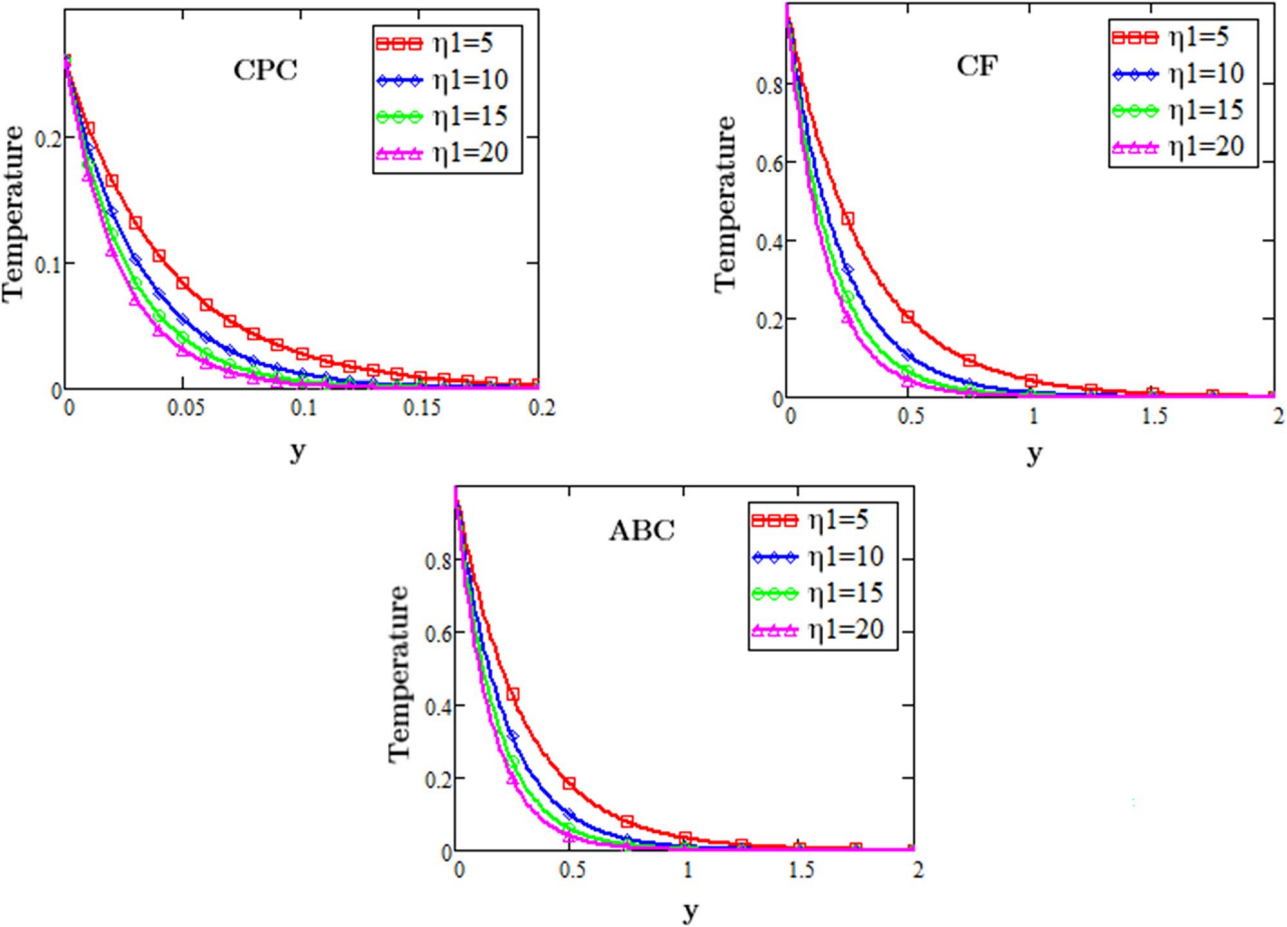
Profiles of heat source for fluid temperature for three different fractional operator.

Figure 1 displays the heat source $\eta_{1}$ effects on second-grade fluid temperature during the flow with the effects of MHD and exponential heating against the space variable y described by three different fractional operator i.e. CF, CPC and ABC derivative operators of fractional order, for numerous values of $\eta_{1}$. From these profiles it is noticed that by rising the value of eta ${\eta }_{1}$ the fluid temperature becomes lower, because the consistency of thermal boundary layer falls with the rising values of parameter eta $\eta_{1}$.

**Figure 2:**
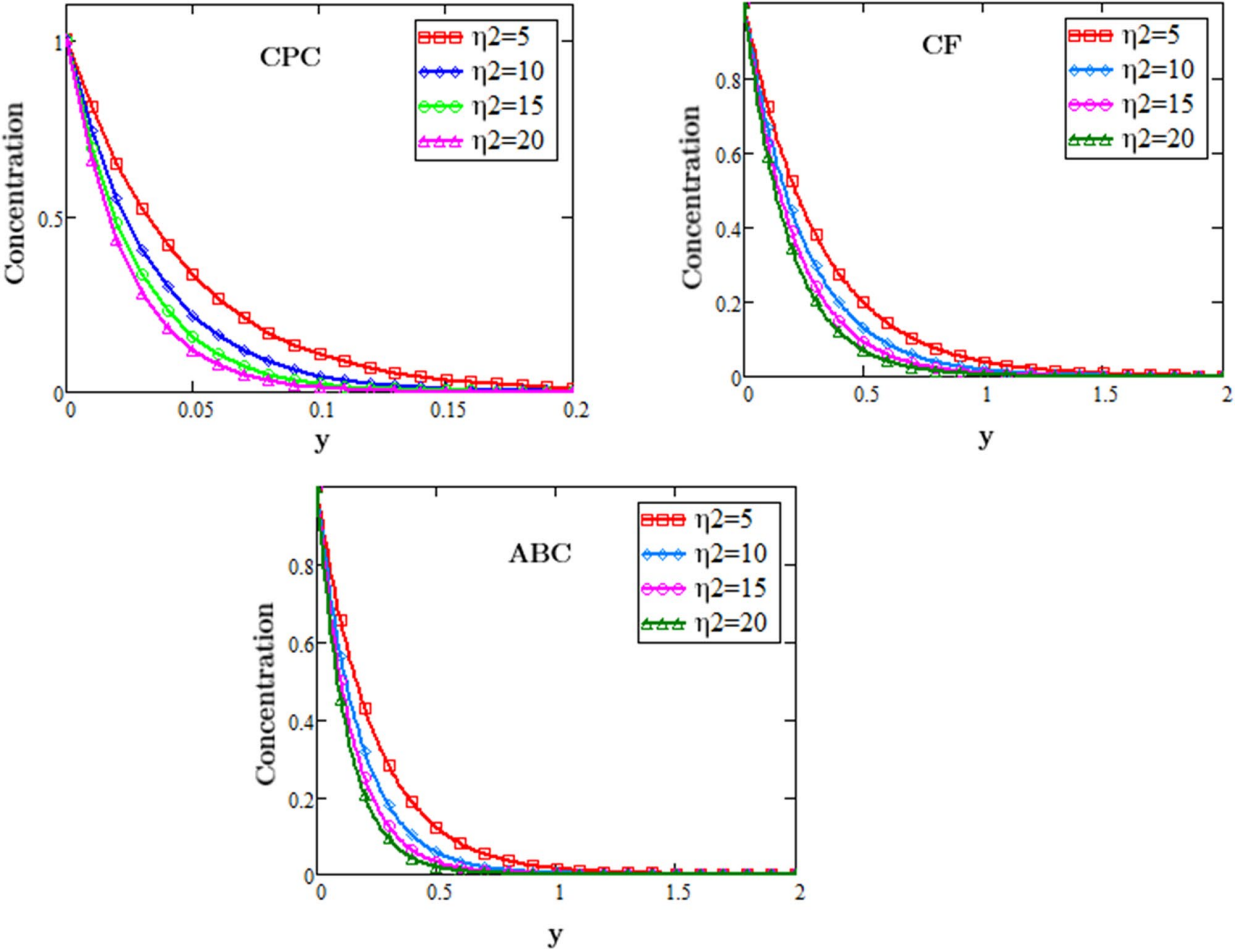
Profiles of chemical reaction for fluid concentration for different fractional operator.

Figure 2 displays the chemical reaction parameter $\eta_{2}$ effects on second grade fluid concentration during the flow influenced by MHD and exponentially changing concentration against the space variable y described by three different fractional operator i.e. CF, CPC and ABC derivative operators of fractional order, for numerous values of $\eta_{2}$. From these profiles it is noticed that by increasing the values of eta $\eta_{2}$ the mass concentration of the fluid decreases, due to the reason when the $\eta_{2}$ values increases it speed ups the reaction rate and when the rate of the reaction increases it increases the conversion of the reactants into the product due to which the concentration decreases.

**Figure 3:**
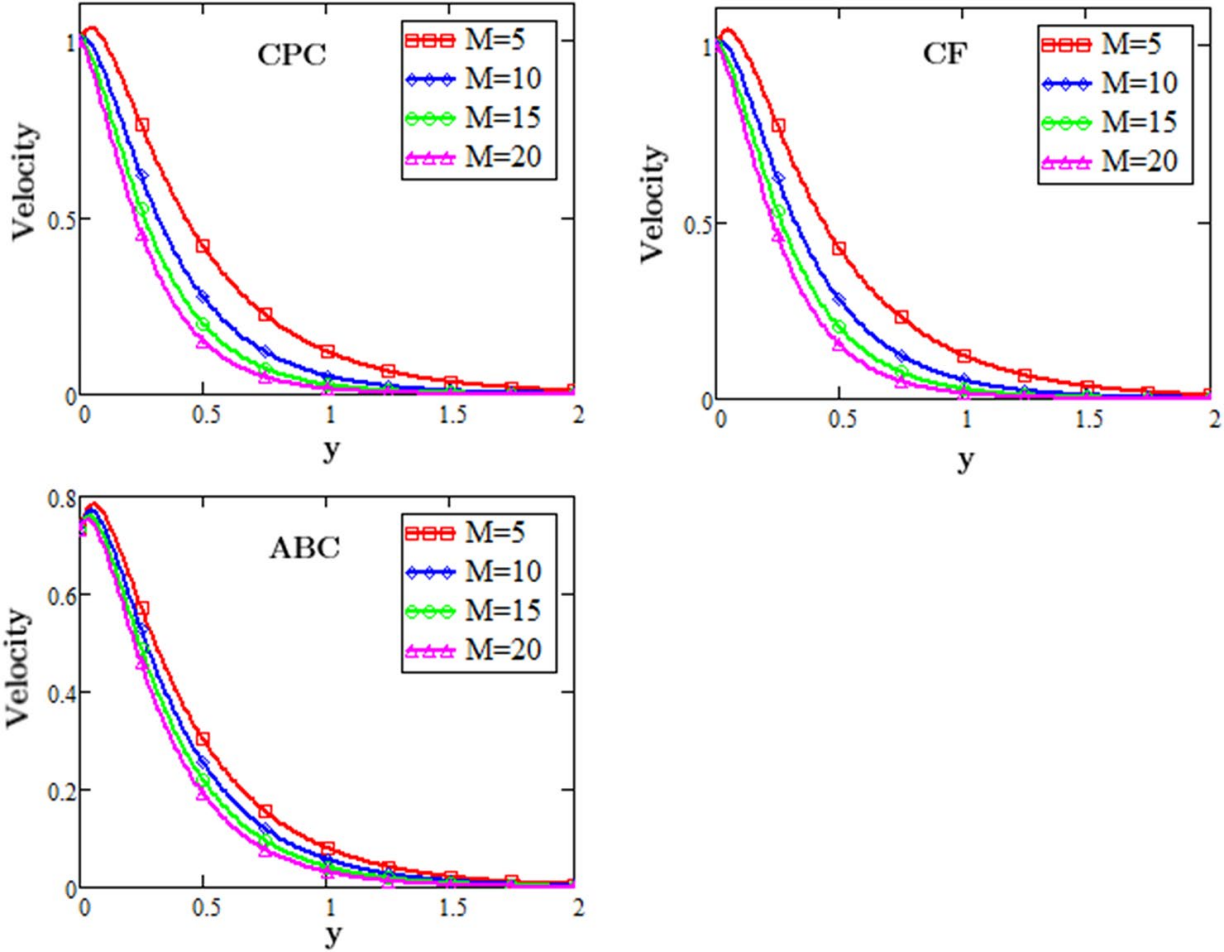
Profiles of MHD for fluid velocity for different fractional operators.

Figure 3 illustrates the behavior of the Magnetic hydrodynamics parameter M effects on second-grade fluid velocity during the flow with the effects of heat source, exponential heating and exponentially changing concentration against the space variable y described by three fractional operator i.e. CF, CPC and ABC, for different values of M. From these profiles it is noticed that by rising the value of Magnetic hydrodynamics parameter M the fluid slows down, because With the increase in the values of the magnetic hydrodynamics results in the increase of the resistive force that is known as “Lorentz force” which increases the drag during the flow due to this increasing drag force causes decrease in the velocity.

**Figure 4:**
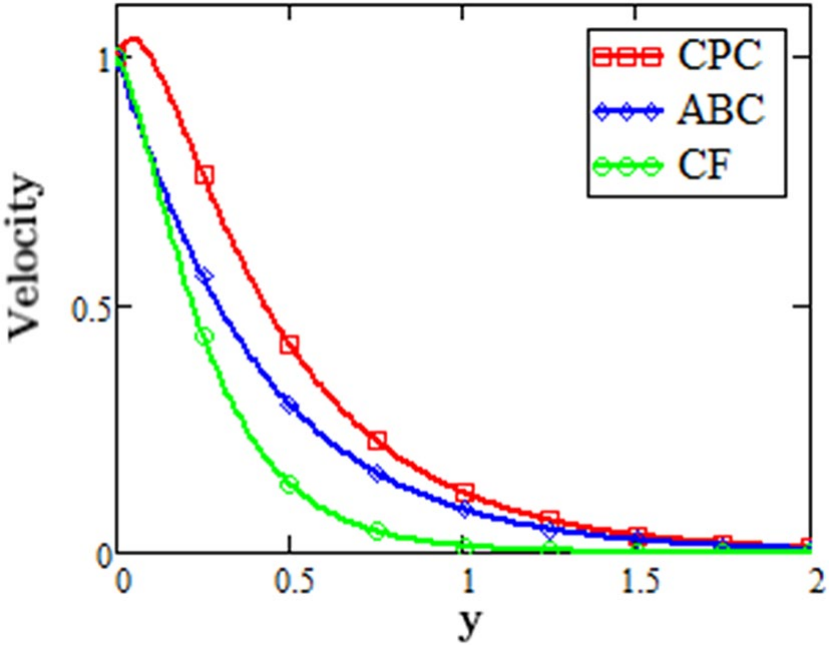
Modification of velocity profile via different fractional parameter.

Figure 4 displays the modification of the fluid velocity obtained from the comparative analysis of the three fractional operators i.e. CF, CPC and ABC. This graph shows that the fluid has the greatest velocity for the fractional operator CPC and the lowest velocity for the fractional operator CF.

## Conclusion

This article provides a comparison among the generalized Second Grade fluid flow described by three different fractional derivatives i.e. CF, ABC and CPC fractional derivative. The heat mass transfer is observed during the flow past a vertical plate that is accelerated exponentially. The effects of the heat generation and exponential heating in the heat boundary layer and chemical reaction at the concentration boundary layer are also analyzed in this article. The fluid is flowing with exponentially variable velocity in a porous medium under the effects of the Magneto hydro dynamics. For the better understanding of the rheological properties of the Second Grade fluid we used the CF, ABC and CPC to interpret the memory effects. The final result (exact analytical) solution of the flow scheme is obtained in the form of G-functions and Mittag Leffler function. For the physical significance of flow parameters, different parameters are graphed. From this analysis it is concluded that the CPC is the most suitable operator to describe the memory effects. Some concluding points are;

- Fluid concentration profile shows decrease with the higher values of $\eta_{2}$ (chemical reaction parameter).
- Fluid temperature profile shows decrease with the rise in the value of $\eta_{1}$ (heat source parameter).
- Fluid velocity profile shows decrease with the increase in the value of MHD parameter.
- Comparison of Fluid velocity profile shows that the motion is faster for the CPC operator as compared to other two.
- From this analysis it is concluded that the CPC is the most suitable operator to describe the memory effects”

### Future recommendations

- The current work can be extended to study of the 3D second grade nanoliquid over a stretching porous surface with melting heat transport mechanism.
- The same problem can be solved with the Mittag-Liffler’s kernel of Prabhakar type fractional derivative.
- The same problem can be solved with the Mittag-Liffler’s kernel of Yang-Abdel- Cattani fractional derivative.

## Data Availability

The datasets used and analyzed during the current study available from the corresponding author on reasonable request.

## List of symbols

$$U_{0} \left[ {LT^{ - 1} } \right]$$: Constant velocity
$$\rho \left[ {L^{2} T^{ - 1} } \right]$$: Density
$$\mu \left[ {ML^{ - 1} T^{ - 1} } \right]$$: Dynamic viscosity
$$\tau \left[ {ML^{ - 1} T^{ - 2} } \right]$$: Shear stress
$$u\left[ {LT^{ - 1} } \right]$$: Velocity
$$T\left[ K \right]$$: Temperature
$$k\left[ {MLT^{ - 3} \theta^{ - 1} } \right]$$: Thermal conductivity
$g\left[ {LT^{ - 2} } \right]$: Acceleration due to gravity
$C_{p} \left[ {L^{2} MT^{ - 1} \theta^{ - 1} } \right]$: Specific heat when pressure is constant
$$y\,\,\left[ L \right]$$: Space coordinates
$$t\,\,\left[ T \right]$$: Time coordinates
$\beta_{T} \left[ {\theta^{ - 1} } \right]$: Thermal expansion coefficient
$\beta_{C} \left[ {L^{ - 3} } \right]$: Mass expansion coefficient
$\gamma$: Parameter of Second grade fluid
$T_{w}$: The temperature of the fluid at the plate
$T_{\infty }$: The fluid temperature at a significant distance from the plate
$$\Pr$$: Prandtl number
$Gr$: Grashof number for thermal
$Gm$: Grashof number for mass
